# MicroRNA responses to focal cerebral ischemia in male and female mouse brain

**DOI:** 10.3389/fnmol.2014.00011

**Published:** 2014-02-11

**Authors:** Theresa A. Lusardi, Stephanie J. Murphy, Jay I. Phillips, Yingxin Chen, Catherine M. Davis, Jennifer M. Young, Simon J. Thompson, Julie A. Saugstad

**Affiliations:** ^1^Dow Neurobiology Laboratories, Legacy Research InstitutePortland, OR, USA; ^2^Department of Anesthesiology and Perioperative Medicine, Oregon Health and Science UniversityPortland, OR, USA

**Keywords:** microRNA, cerebral ischemia, sex-differences, array analysis, qRT-PCR, pathway analysis, stroke

## Abstract

Stroke occurs with greater frequency in men than in women across diverse ethnic backgrounds and nationalities. Work from our lab and others have revealed a sex-specific sensitivity to cerebral ischemia whereby males exhibit a larger extent of brain damage resulting from an ischemic event compared to females. Previous studies revealed that microRNA (miRNA) expression is regulated by cerebral ischemia in males; however, no studies to date have examined the effect of ischemia on miRNA responses in females. Thus, we examined miRNA responses in male and female brain in response to cerebral ischemia using miRNA arrays. These studies revealed that in male and female brains, ischemia leads to both a universal miRNA response as well as a sexually distinct response to challenge. Target prediction analysis of the miRNAs increased in male or female ischemic brain reveal sex-specific differences in gene targets and protein pathways. These data support that the mechanisms underlying sexually dimorphic responses to cerebral ischemia includes distinct changes in miRNAs in male and female brain, in addition to a miRNA signature response to ischemia that is common to both.

## Introduction

Stroke occurs more frequently in men than women across diverse ethnic backgrounds and nationalities (Bushnell, 2008; Reeves et al., 2008; Saini and Shuaib, 2008; Appelros et al., 2009; Persky et al., 2010; Ovbiagele et al., 2013; Towfighi et al., 2013). Our lab and others have shown that sensitivity to cerebral ischemia, i.e., the extent of brain damage resulting from ischemic insult, is sex-specific, with female animals being less sensitive than males (Murphy et al., 2004; Koerner et al., 2007; Lang and McCullough, 2008; Reeves et al., 2008; Vagnerova et al., 2008; Cheng and Hurn, 2010; Siegel et al., 2010). Sex-specific responses are also observed in response to focal cerebral ischemia in isoflurane preconditioned mice (Kitano et al., 2007) and in immune responses to ischemia (Banerjee et al., 2013). Furthermore, sex differences in ischemic sensitivity have been extended to the cellular level as our lab and others have shown that astrocytes (Liu et al., 2007, 2008) and neurons (Li et al., 2005; Johnsen and Murphy, 2011) from male newborn rodents are more sensitive to oxygen-glucose deprivation (an *in vitro* model of ischemia) than cells from female newborn rodents. These observations suggest that the male brain exhibits a more “ischemia-sensitive” phenotype than the female brain. However, the underlying molecular mechanisms for this sexually dimorphic response to ischemia are not well understood.

We examined a role for miRNAs in ischemic responses in the male and female brain. MiRNAs are short, non-coding RNA sequences that regulate post-transcriptional gene expression via translational repression or mRNA degradation (Ambros, 2004; Murchison and Hannon, 2004; Niwa and Slack, 2007; Guarnieri and DiLeone, 2008; Chua et al., 2009). MiRNAs have been implicated in the regulation of numerous physiological and pathological processes such as brain differentiation (Feng and Feng, 2011), neurological disorders (Saugstad, 2010), ischemic preconditioning (Lusardi et al., 2010), and stroke (Rink and Khanna, 2011; Tan et al., 2011). The few studies which have examined miRNA responses to injury in brain have either focused on irradiation injury (Ilnytskyy et al., 2008; Koturbash et al., 2011), evaluated a single miRNA target of interest following brain ischemia (Siegel et al., 2011), or profiled miRNAs in male ischemic brain without linking them functionally to ischemic mechanisms and outcomes (Jeyaseelan et al., 2008; Dharap et al., 2009; Liu et al., 2010; Lusardi et al., 2010).

For these studies we focused on miRNA expression at 8 h after ischemia, based on our previous miRNA studies in rodent brain showing that this reperfusion time is optimal for robust change in miRNA expression levels. Two previous studies revealed little or no changes in miRNA expression at 2 and 4 h after treatment, robust changes 8 h after treatment, and a return to levels comparable to naïve controls by 24 h after treatment (Lusardi et al., 2010, 2012). These studies suggest that the treatments used (ischemia or glutamate activation) induced transcriptional changes in miRNA expression, or alterations in the miRNA processing pathway, that were optimally detected 8 h after the treatment. This time course would be consistent with miRNAs as early mediators of mRNA translation and protein expression that in turn lead to cellular changes that develop within 24–72 h after ischemia.

Our miRNA profiling studies revealed that there are sex-specific differences in miRNA responses to ischemia as well as a universal, ischemia-induced miRNA signature equally present in both male and female brains. Our findings reveal a novel mechanism, namely the differential regulation of miRNA responses, for sex differences in ischemic sensitivity mediated by sex-specific miRNA pathways in male and female brain.

## Materials and methods

### Experimental groups

Experiments were carried out in male and female C57BL/6 mice (Charles River Laboratories, Wilmington, MA, USA), 8–14 weeks of age and weighing 20–25 g. Experiments were carried out in accordance with the National Institutes of Health guidelines for research animal care and approved by the Oregon Health and Science University Animal Care and Use Committee. All mice were maintained on a 12/12 h light-dark cycles and permitted *ad libitum* access to food and water. Male and female mice were randomized to one of the following experimental groups: control (experimentally naïve), sham surgery, or transient focal cerebral ischemia.

### Transient focal cerebral ischemia

All surgeries were conducted under aseptic conditions by a single surgeon. Transient focal cerebral ischemia was induced in male and female mice for 60 min by reversible right middle cerebral artery occlusion (MCAO) under isoflurane anesthesia, followed by 8 h of reperfusion as previously described (Chen et al., 2012). Peri-ischemic head and body temperature were controlled at 36.5 ± 1.0°C (mean ± standard deviation) with warm water pads and a heating lamp. The common carotid artery was temporarily occluded while a 6-0 nylon monofilament surgical suture (ETHICON, Inc., Somerville, NJ, USA) with a silicone-coated (Xantopren Comfort Light, Heraeus Kulzer, Germany) tip was inserted via an external carotid artery stump distal to the internal carotid artery to the origin of the middle cerebral artery. After 60 min of MCAO, the filament was withdrawn to allow for reperfusion. All incisions were the closed with 6-0 surgical sutures (ETHICON, Inc., Somerville, NJ, USA) before each mouse was awakened and recovered in a separate cage with a warm water pad. For sham surgeries, the filament was placed but not advanced to achieve MCAO. Occlusion and reperfusion were verified in each mouse by laser Doppler flowmetry (LDF) (Model DRT4, Moor Instruments Inc. Wilmington, USA). Mice were excluded if intra-ischemic LDF (% pre-ischemic LDF baseline) was greater than 25%. Neurological deficit scores were determined at 1 h of reperfusion to confirm the presence of ischemic injury using a 0–4 point scale as follows: 0, no neurological dysfunction; 1, failure to extend left forelimb fully when lifted by tail; 2, circling to the contralateral side; 3, falling to the left; and 4, no spontaneous movement or in a comatose state (Chen et al., 2012). Any animal without a deficit at 1 h of reperfusion was excluded from the study. Eight hours following either sham surgery or focal cerebral ischemia, mice were anesthetized with isoflurane and euthanized by decapitation. Experimentally naïve mice were also anesthetized with isoflurane and euthanized by decapitation. Right and left cortices were sub-dissected from each mouse brain, and the tissues were frozen in 2 methyl-2-butane on dry ice, then stored at −80°C.

### RNA isolation

To correlate with the right MCAO model, RNA was isolated from the right mouse brain cortex with the mirVana miRNA Isolation Kit (Life Technologies, Carlsbad, CA, USA), following the recommended protocol for total RNA isolation from frozen tissue. The RNA isolation did not include the “Enrichment Procedure for Small RNA” in the protocol provided with the kit. Total RNA was eluted with 100 μ L of Elution Solution provided with the RNA isolation kit, and the RNA concentrations quantified by spectroscopic measurement of A260. RNA samples were stored at −80°C until further use.

### MicroRNA array profiling

Mouse MicroRNA Genome V2.0 PCR Arrays (MAM-200C; SABiosciences/Qiagen, Valencia, CA) were used to quantitatively assay miRNA expression in mouse brain. The arrays consisted of the 528 most abundantly expressed and well-characterized miRNA sequences in the mouse genome, as annotated by the Sanger miRBase Release 14. For qRT-PCR array analysis we used total RNA samples representing control male and female mice, and ischemic male and female mice. The RT2 miRNA First Strand Kit (SABiosciences) was used for Reverse Transcription (RT) of the RNAs, as per the manufacturer's instructions. Total RNA from control and ischemic male and female mice (*n* = 3 mice/group) were pooled, then 2 μ g of the pooled total RNA was incubated in RT buffers at 37°C for 2 h, followed by 95°C for 5 min to degrade the RNA and to inactivate the reverse transcriptase. The first-strand cDNA samples were chilled on ice then diluted with RNase-free water. The RT2 SYBR green master mix (PA-012) was used for the qRT-PCR reactions, as per the manufacturer's instructions (SABiosciences). Briefly, the diluted first-strand cDNA was combined with master mix, then 25 μL was aliquoted into each well of six 96 well plates. The six plates were briefly centrifuged then stored at −20°C. For amplification, individual plates were removed from −20°C, defrosted for 5 min at RT, briefly centrifuged and placed into a 7500 Fast Real-Time PCR System (Applied Biosystems, Foster City, CA). Parameters were set to: (i) one cycle at 95°C for 10 min, (ii) 40 cycles at 95°C for 15 s, 60°C for 40 s, and 72°C for 30 s. A dissociation step set to: (iii) 95°C for 15 s; 60°C for 1 min and 95°C for 15 s was performed to ensure that all PCRs generated a single product. Normalized delta CT (ΔCt) values were calculated with respect to the average of all Ct values, and ΔΔCt was calculated as (ΔCt-Ischemic minus ΔCt-Control) for all of the male and female miRNAs.

### Sabiosciences qRT-PCR microRNA array normalization

We calculated the ΔCt for each miRNA in a given experiment based on the average Ct value of all miRNAs in that experimental condition: ΔCtmir = Ctavg − Ctmir. We then determined the response to ischemia for males and females separately, and calculated the change in miRNA expression as ΔΔCtmir = ΔCtmirischemia − ΔCtmircontrol. We defined statistical significance as a multiple of a standard deviation from the mean ΔΔCt (SD).

### Real-time qRT-PCR detection

We analyzed miRNA expression in individual mice (each group) from control (*n* = 5), sham (*n* = 5), and ischemic (*n* = 4) cortices. Sham surgery groups were added after the initial miRNA array profiling to account for any effects due to anesthesia and surgical stress. Detection of miRNAs was completed with a 2-step qRT-PCR assay, using the miScript PCR System (Qiagen, Valencia, CA). One microgram of total RNA was converted to cDNA, for RNA from each individual sample, with the miScript II RT Kit, following the standard protocol with the miScript HiSpec Buffer. Negative control samples included those with no reverse transcriptase (No RT). The qRT-PCR assays were prepared for each individual using 1 μ g of total RNA in the same reverse transcription reaction protocol, omitting the RT enzyme in the reaction mix. The resulting 20 μ L of RT products (or No RT controls) were diluted to a total volume of 100 μ L with the addition of 80 μ L RNase/ DNase Free water, and stored at −80°C before use in PCR assays. Two micro litres of cDNA was assayed in each PCR reaction well. miScript PCR Primer Assays (Qiagen) were used for detection of mature miRNA, using the miScript SYBR Green PCR Kit for qRT-PCR assays. The following primer sets were used:

**Table:** 

miR-15b	5′ CGAAUCAUUAUUUGCUGCUCUA	(#MS00011242)
miR-125b-3p	5′ ACGGGUUAGGCUCUUGGGAGCU	(#MS00024066)
miR-296-5p	5′ AGGGCCCCCCCUCAAUCCUGU	(#MS00016436)
miR-509-3p	5′ UGAUUGACAUUUCUGUAAUGG	(#MS00012306)
miR-682	5′ CUGCAGUCACAGUGAAGUCUG	(#MS00033019)
miR-686	5′ AUUGCUUCCCAGACGGUGAAGA	(#MS00002821)
miR-883a-3p	5′ UAACUGCAACAGCUCUCAGUAU	(#MS00012845)
miR-883b-3p	5′ UAACUGCAACAUCUCUCAGUAU	(#MS00012859)
miR-1224	5′ GUGAGGACUGGGGAGGUGGAG	(#MS00011074)

Assays were performed with the standard recommended reaction mix (25 μ L volume per reaction) in 96-well reaction plates, using a ViiA 7 Real Time PCR Detection System (Life Technologies). Each miRNA assay was performed in triplicate for individual samples. The Ct values were calculated using the same cycle threshold and baseline for all reactions. The ΔCtmir = Ctnon-changers − Ctmir, responses to ischemia for males and females were calculated the change in miRNA expression as ΔΔCtmir = ΔCtmirischemia − ΔCtmircontrol, and ΔCtmirischemia − ΔCtmirsham.

### MicroRNA target prediction

We used the miRmap target prediction program (http://mirmap.ezlab.org/, accessed November 2013) to query the predicted targets of miRNAs increased by ischemia in male and female brain (Vejnar et al., 2013). We then used the predicted protein targets identified in miRmap to query the PANTHER v8.1 program (http://www.pantherdb.org/, accessed November 2013). The PANTHER (Protein ANalysis THrough Evolutionary Relationships) classification system was designed to classify proteins (and their genes) according to: family and subfamily, molecular function, biological process, and pathway that explicitly specifies the relationships between the interacting molecules (Mi and Thomas, 2009; Mi et al., 2013).

## Results

### Statistical analysis of microRNA arrays

**Figure 1:**
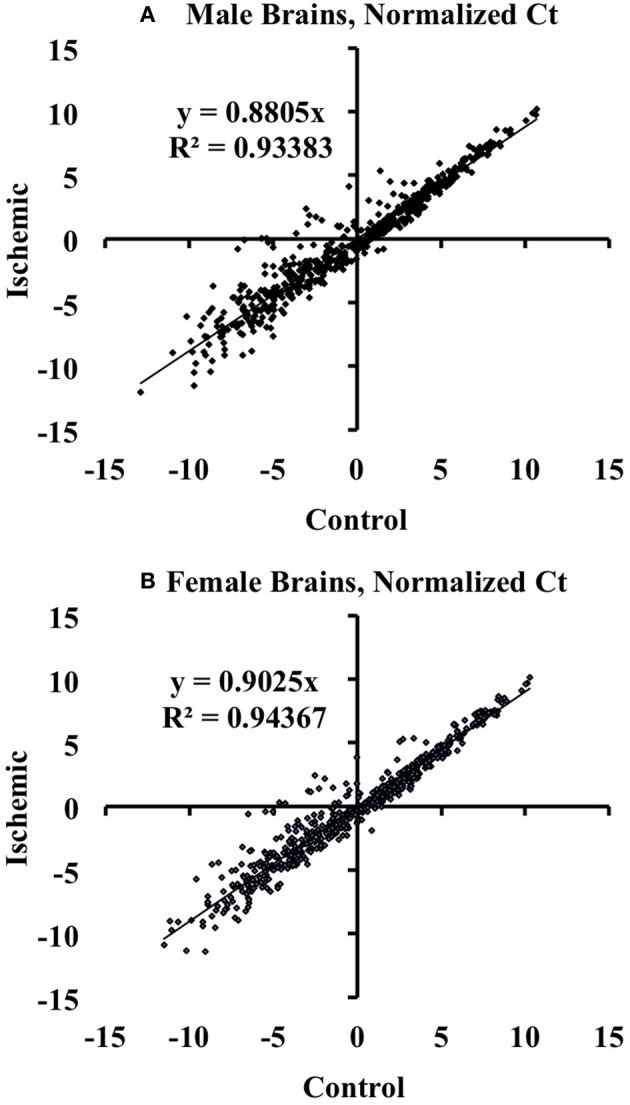
**Linear regression analysis of miRNAs altered by ischemia vs. control mouse cortices**. The graphs illustrate that there are changes in miRNA expression in ischemia relative to control in male **(A)** and female **(B)** mouse cortex. The graphs also show that the majority of the assayed miRNAs are not altered by ischemia.

We used the Mouse MicroRNA Genome V2.0 PCR Arrays to profile the expression of mouse cortical miRNAs in both male and female brains. For the array studies, we pooled RNA samples isolated from control and ischemic male and female cortices. For array normalization, we first considered the endogenous controls included on the PCR arrays (Snord85, Snord68, Snord66, and Rnu6). We found that within a given treatment group, the endogenous controls were similar from plate-to-plate. However, across experimental groups the response of the endogenous controls was not consistent for sex or stroke groupings. We therefore calculated the ΔCt for each miRNA in a given experiment (control or ischemia) based on the average Ct value of all miRNAs in that experimental condition: ΔCtmiR = Ctavg − CtmiR, consistent with the assumption that most miRNAs would not be altered by focal ischemia as shown by linear regression analysis in Figure 1.

**Figure 2:**
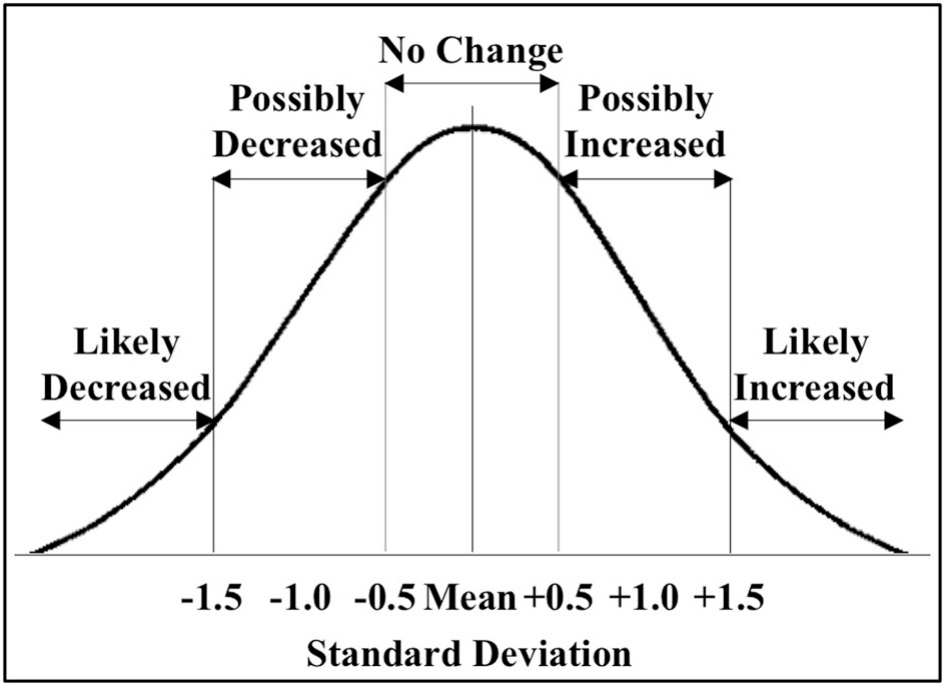
**Classification of miRNAs altered by ischemia**. The figure shows the distribution of the miRNAs altered by ischemia relative to control in male and female mouse cortex. For initial studies we focused on miRNAs that were significantly decreased (<−1.5 *SD*) or increased (>1.5 *SD*) in the male and female mouse cortex.

We then determined the response to ischemia for males and females separately, and calculated the change in miRNA expression as ΔΔCtmiR = ΔCtmiRischemia − ΔCtmiRcontrol. We defined significance as a multiple of a standard deviation from the mean ΔΔCt (SD), as shown in Figure 2. A miRNA was considered to be significantly decreased in response to ischemia if ΔΔCtmir (≤−1.5 *SD*); the specific miRNAs are listed in Table 1A. A miRNA was considered to be significantly increased in response to ischemia if ΔΔCtmir (≥1.5 *SD*); specific miRNAs are listed in Table 1B. Approximately half of the 528 miRNAs did not change in response to ischemia based on the cutoff criterion (−0.5 *SD*) < ΔΔCtmir < (0.5 *SD*). An additional group of miRNAs possibly changed in response to ischemia, with a (0.5 *SD*) < |ΔΔCtmir| < (1.5 × *SD*), but did not meet the criterion for significance and were excluded from further analysis in this study.

**Table 1:** **MicroRNAs Regulated By Ischemia**.

**Male**	**ΔΔCt**		**Male and Female**	**ΔΔCt**		**Female**	**ΔΔCt**	
	**M**	**F**		**M**	**F**		**M**	**F**
**(A) SIGNIFICANTLY DECREASED IN ISCHEMIA (<−1.5 *SD*)**								
miR-19a^*^	−2.57	−0.23	**miR-296-5p**	−2.16	−2.76	***miR-509-3p***	*2.03*	−2.35
miR-145^*^	−2.68	−1.41				miR-543	−1.70	−1.79
miR-450b-3p	−2.32	0.22				miR-741	−0.22	−1.87
***miR-883b-3p***	−2.37	2.18						
**(B) SIGNIFICANTLY INCREASED IN ISCHEMIA (>1.5 *SD*)**								
miR-207	2.22	1.64	miR-27a^*^	4.06	3.94	miR-197	1.30	2.47
miR-218-2^*^	2.37	1.06	miR-135a^*^	3.95	2.56	miR-200c^*^	1.51	1.95
miR-327	3.17	1.57	miR-196a^*^	2.79	1.93	miR-466f-3p	1.58	2.03
miR-345-3p	2.26	0.76	miR-200a^*^	2.59	2.13	miR-466f-5p	1.06	2.08
miR-466g	2.33	1.29	miR-200b^*^	2.72	2.10	miR-615-3p	0.18	2.47
miR-493	4.33	1.59	miR-291a-5p	3.00	2.84	miR-875-3p	1.43	2.29
***miR-509-3p***	2.03	−2.35	miR-323-5p	2.01	2.94	**miR-883a-3p**	−0.83	1.86
miR-669g	2.47	0.00	miR-370	4.57	3.86	***miR-883b-3p***	−2.37	2.18
miR-675-3p	2.90	0.73	miR-466i	1.98	1.79	miR-883b-5p	0.93	2.88
**miR-682**	2.12	−0.41	miR-470^*^	3.30	2.37			
miR-697	2.27	−0.09	miR-483^*^	4.84	3.70			
miR-770-5p	2.10	1.33	miR-546	2.03	2.32			
miR-1187	2.16	0.20	miR-681	1.95	2.16			
miR-1190	2.66	1.12	miR-615-5p	3.40	2.22			
miR-1892	3.06	1.22	miR-654-3p	4.03	3.88			
miR-1896	2.18	0.70	miR-677	2.78	4.10			
miR-1897-5p	1.97	0.02	miR-678	2.87	2.60			
			miR-684	2.03	3.86			
			miR-685	5.74	4.60			
			**miR-686**	6.32	5.88			
			miR-695	2.95	1.77			
			miR-709	2.50	2.59			
			miR-712^*^	5.43	4.97			
			miR-743b-5p	2.17	2.42			
			miR-1188	4.67	4.11			
			miR-1195	4.07	4.15			
			miR-1196	4.98	4.56			
			miR-1199	5.41	4.95			
			**miR-1224**	4.17	3.67			
			miR-1895	3.58	2.46			
			miR-1897-3p	6.67	5.09			

*MiRNAs significantly regulated by ischemia in male and female mouse brain*.

***(A)** Lists the ΔΔCts for miRNAs significantly decreased in ischemia (<−1.5 SD) in male and female cortex*.

***(B)** Lists the ΔΔCts for miRNAs significantly increased by ischemia (>1.5 SD) in male and female cortex*.

*The bold indicates the miRNAs in the table that were used for subsequent studies, the data for which is presented in Figure 4. The italics indicate those miRNAs that were found to be oppositely regulated in male and female brain*.

For initial studies, miRNA was considered significantly changed if ΔΔCt was greater than 1.5 *SD* (standard deviation) from the mean ΔΔCt. The studies revealed two profiles: (1) sex-dependent responses wherein ischemia-regulated miRNAs were unique to male or female brain, and (2) a sex-independent response in which miRNAs are equally present and equally regulated in both male and female brains (the focus of another study). Of the significantly increased miRNAs, >50% were present in both male and female brain (Table 1).

### Individual microRNA validation

Individual miRNAs representing down- and up-regulated species in male and female mice were further validated by Taqman qRT-PCR assays (Qiagen). Although the initial miRNA array studies did not include a sham surgery group, we did include sham surgery groups in the individual qRT-PCR validation studies to account for any treatment effects due to anesthesia and surgical stress. As indicated in Table 1, we examined miR-883b-3p that decreased in males and increased in females, miR-296-5p that decreased in males and females, miR-509-3p that decreased in females (1A). We also examined miR-682 that increased in males, miR-686 and miR-1224 that increased in males and females, and miR-883a-3p that increased in females (1B). Bold font in Table 1 highlights the miRNAs that were chosen for individual qRT-PCR validation, while italic font indicates the miRNAs that showed significant changes in the opposite direction in male and female brain.

**Figure 3:**
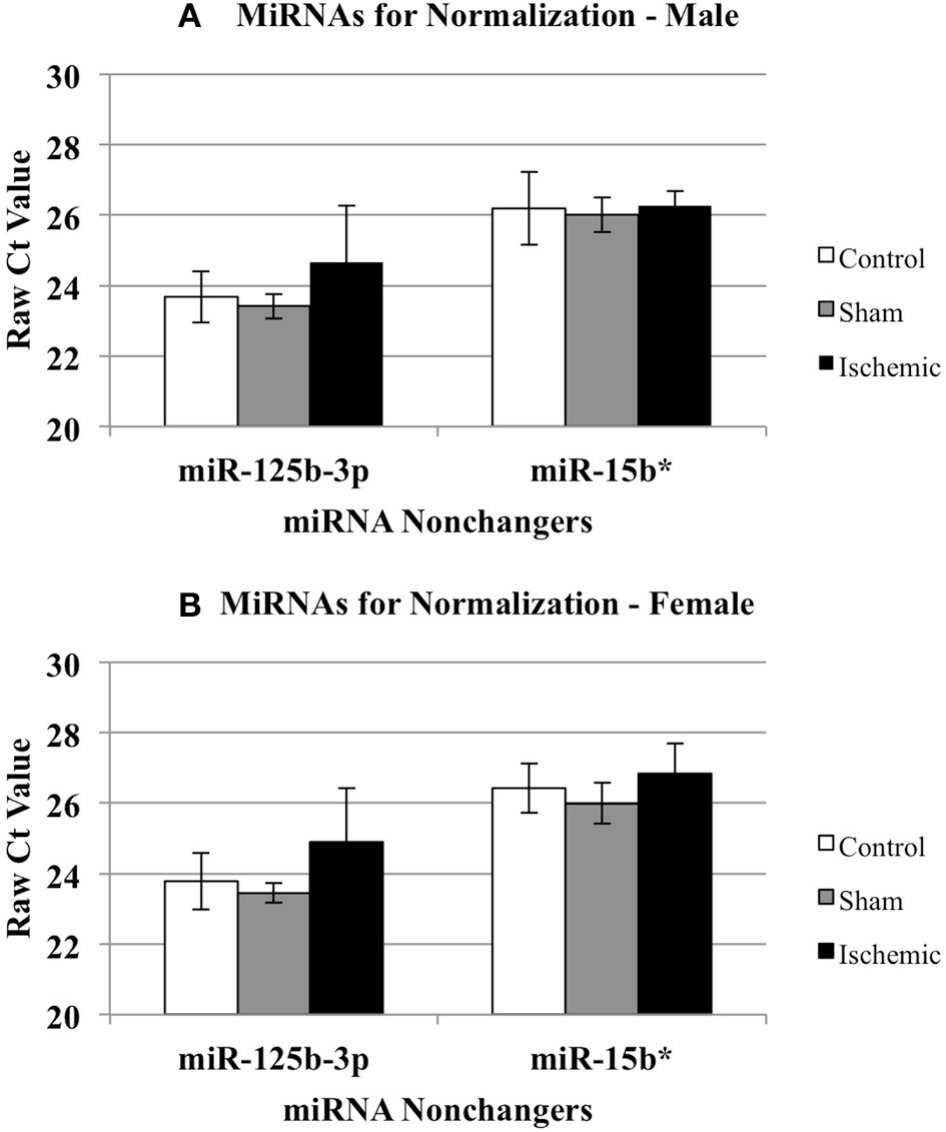
**Effect of treatment on miRNAs identified as non-changers in male and female mouse cortex**. The graphs illustrate that miR-15b^*^ and miR-125b-3p were not changed significantly in any treatment group in male **(A)** or female **(B)** mouse cortex. Control (white bar), sham (gray bar), ischemia (black bar).

We calculated the ΔΔCts using both control (*n* = 5 each male and female) and sham surgery (*n* = 5 each male and female) ΔCt values, relative to ischemia (*n* = 4 each male and female). Based on the array results, we selected miR-15b^*^ and miR-125b-3p as candidates for a normalizing factor for our qRT-PCR validation. The rationale for this choice is as follows. We compared the raw Ct values for each of these miR across experimental conditions (control, sham, and ischemic) using a 2-Way repeated measures ANOVA (Prism, GraphPad Software, Inc. La Jolla, CA). The results showed: (1) there is no effect of the experimental condition (i.e., ischemia vs. sham vs. control) (*p* = 0.1429), there is a significant difference between miR-15b^*^ and miR-125b-3p (*p* < 0.0001), and (3) there was no interaction between the experimental condition and the miRNAs (*p* = 0.1272). The within subject matching is significant (*p* = 0.0004), suggesting that variation across is due to individual variation, not to chance. These analyses support that neither miR-125b-1-3p nor miR-15b^*^ is sensitive to the experimental manipulations (Figure 3) in males (3A) or females (3B), thus the average of both was used as a normalizing factor.

**Figure 4:**
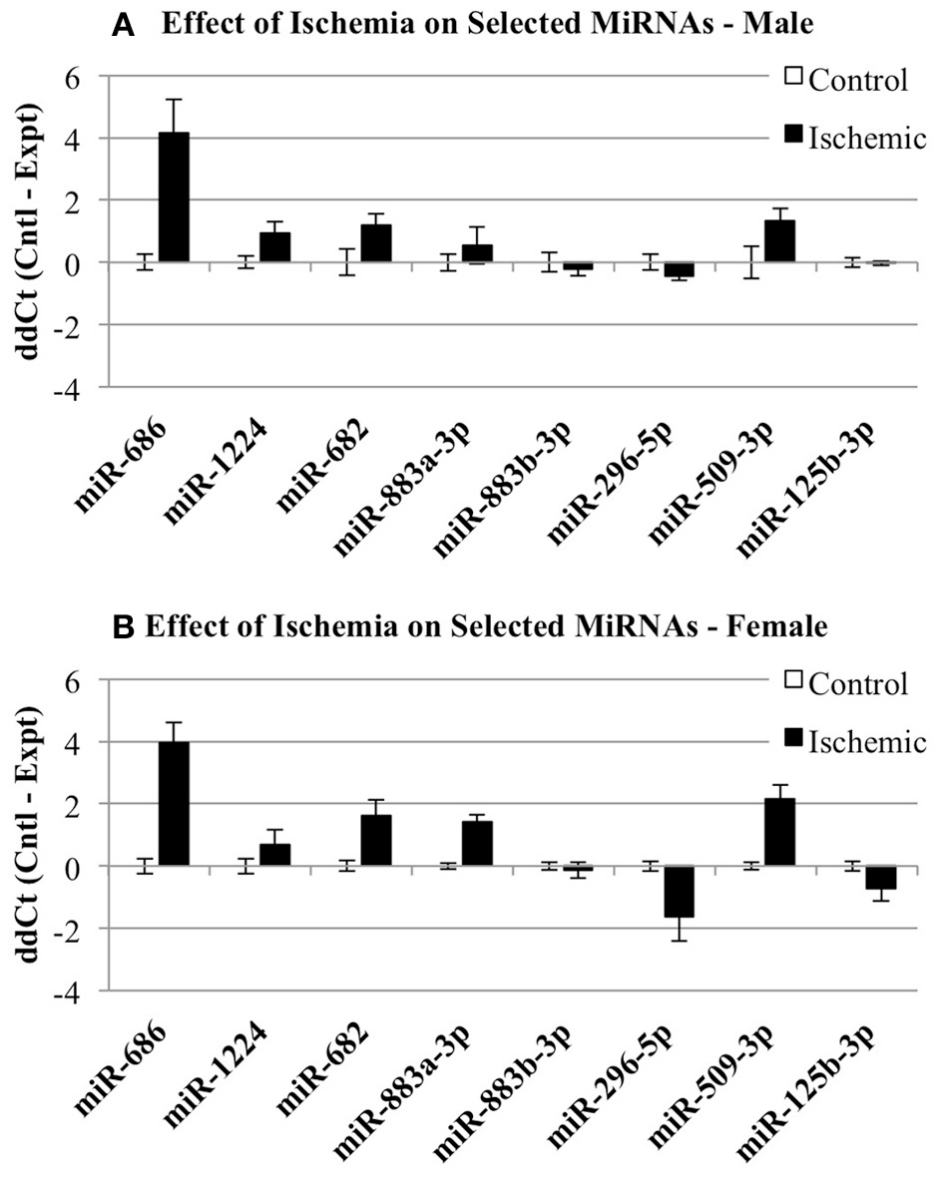
**Individual microRNAs regulated by ischemia in male and female mouse cortex**. The graphs illustrate the ΔΔCt changes for specific miRNAs chosen for further validation by individual qRT-PCR. The graphs show expression of miRNAs in control (white bar) and ischemic (black bar) male **(A)** or female **(B)**.

We then examined whether any of the selected miRNAs were sensitive to the sham surgery. Since the original miRNA array comparison was performed on control vs. ischemic conditions, we examined whether there was any significant influence of a surgical sham on miRNA expression. Thus, we compared the normalized Ct values (ΔCt) for each miRNA in Control and Sham experimental groups using a 2-Way repeated measures ANOVA (Prism, GraphPad Software). The results show: (1) there is no effect of the experimental group (*p* = 0.1130), (2) there is a significant effect of miRNA (*p* < 0.0001), (3) there is no interaction between miRNA and experimental group (*p* = 0.1344), and (4) there is a significant within-subject matching (*p* = 0.0116), suggesting variations are due to individual subject variation, rather than random. The conclusions from these analyses are that there is no influence of the surgery alone on any of the miRNAs examined here. Thus, Figure 4 shows the changes in select miRNAs expressed in male (Figure 4A) of female (Figure 4B) cortex in control vs. ischemic treated cortex.

**Figure 5:**
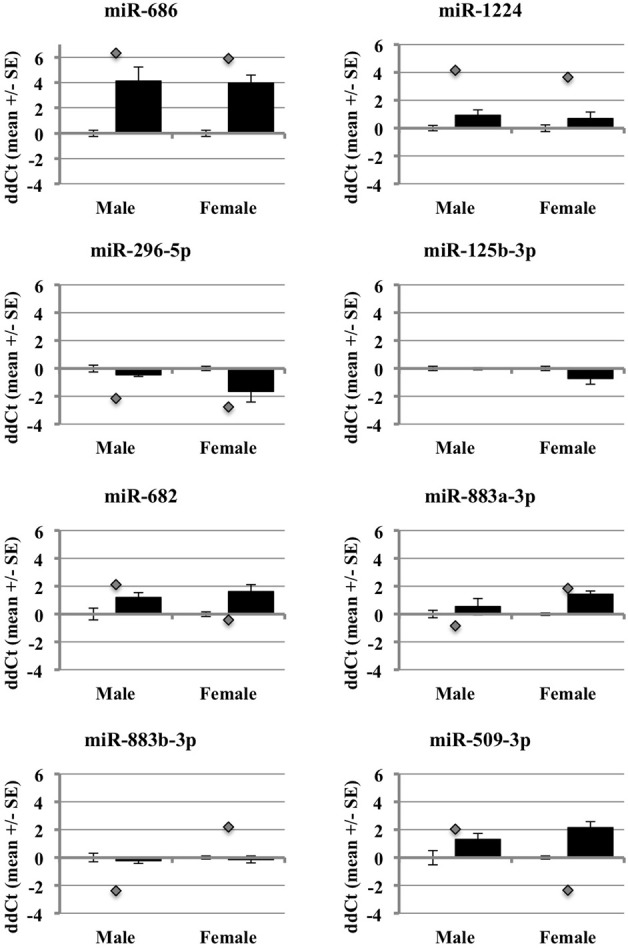
**Comparison of miRNA array expression to individual qRT-PCR assays in male and female mouse cortex**. The graphs show the ΔΔCt of male and female miRNAs control (white bar) or ischemic (black bar) mouse cortex. The graphs also include the ΔΔCt for each miRNA as detected in the miRNA arrays (gray diamond).

After accounting for the above, the results show that many but not all of the miRNAs under study showed changes in the same direction (up or down) in the individual qRT-PCR assays, consistent with the changes in expression detected by the pooled samples in the miRNA arrays (Figure 5). MiR-686 and miR-1224 both showed increased expression in males and females, as predicted by the miRNA arrays. In addition, miR-296-5p decreased in males and females, and miR-125b-3p showed no changes in male or female brain, consistent with the miRNA arrays. MiR-682 showed increased expression in males, consistent with the miRNA arrays, but there was increased expression in females that was opposite from the arrays. MiR-883a-3p showed increased expression in females, consistent with the miRNA arrays, but there was a slight increase in the expression in males relative to the small decrease in expression that was detected in the arrays. Also, miR-509-3p increased in the males, consistent with the miRNA arrays, but decreased significantly in the females which was opposite from the arrays.

These outcomes support that miRNAs are regulated by ischemia in male and female brain. However, they also show that the use of different array formats, or pooled vs. individual qRT-PCR samples, likely influences the expression results (Git et al., 2010). The miRNAs with the most robust changes in expression in the arrays showed consistent changes in the individual arrays, particularly for those miRNAs that increased in expression. This observation suggests that decreases in miRNA expression from RNA degradation, low abundance of miRNAs, or pooling samples, may affect the array outcomes and data interpretation.

### MicroRNA target prediction

We used the miRmap target prediction program (http://mirmap.ezlab.org/) to query the predicted targets of miRNAs increased by ischemia in male and female brain (Vejnar et al., 2013). We used miRmap to query the targets of the 17 miRNAs increased by ischemia in male brain. The results show target genes and the top number of miRNAs predicted to target this gene with a cutoff of 6 (Table 2A). We also used miRmap to query the targets of the 9 miRNAs increased by ischemia in female brain. The results show target genes and the top number of miRNAs predicted to target this gene with a cutoff of 4 (Table 2B). The results show that the miRNAs increased by ischemia in male and female brain are distinct, and only 2 targets are detected in common in the groups, CD73 and PKN2. These findings suggest that differential pathways are targeted by miRNAs increased by ischemia and that these pathways may lead to differential outcomes to ischemia may underlie the sexually dimorphic responses to ischemia, wherein females are afforded greater protection against ischemia insult than males. PANTHER target analysis revealed that there are 36 pathways of the genes targeted by miRNAs increased in male brain, and there are 69 pathways for the genes targeted by miRNAs in the female brain (Table 3—Supplemental Data). These findings support that distinct pathways are targeted by miRNA responses to ischemia, and provide an opportunity to focus studies on specific protein and protein pathways that may have been overlooked in prior studies.

**Table 2:** **Predicted Proteins Targeted By MicroRNAs Significantly Increased Following Ischemia**.

**(A) Male**		**(B) Female**			
**Of 17 Increased microRNAs**		**Of 9 Increased microRNAs**			
**Gene**	**No. of microRNAs**	**Gene**	**No. of microRNAs**	**Gene**	**No. of microRNAs**
Brwd3	9	Cdc73	6	Lyvel	4
Ccr3	7	Cnrl	6	Mbtps2	4
Cdc73	7	Cadm2	5	Mklnl	4
Clintl	7	Cbln2	5	Mon2	4
Em15	7	Lcorl	5	Naa50	4
Fam55c	7	Pkn2	5	Nucksl	4
Map3k2	7	S1c30a4	5	Odzl	4
Mef2c	7	S1c6a14	5	Ogfrll	4
Ano3	6	Slitrk4	5	Prrg3	4
Atp 1 3a3	6	Strbp	5	Pura	4
Atp2c1	6	Taf4 a	5	Ragl	4
Ccdc93	6	Akap2	4	Rbpms2	4
Cnot61	6	Ap4e 1	4	Rhoa	4
Coll lal	6	Arl5b	4	Rqcdl	4
Csmdl	6	Atadl	4	Sec24a	4
Dio2	6	Atpafl	4	Serpinb7	4
Edaradd	6	Cblb	4	Slitrkl	4
Eif2c3	6	Cd200r4	4	Sox4	4
Fgfbp3	6	Clec2h	4	Sox5	4
Gabrg3	6	Cltc	4	Syngr3	4
Ghr	6	Crebzf	4	Tbc1d24	4
Gng2	6	Cxxc4	4	Tdrd5	4
Hipk3	6	Ect2	4	Tecrl	4
Hmgcsl	6	Femlb	4	Tmem236	4
Htrl a	6	Flil	4	Trip 12	4
Illrapll	6	Foxal	4	Ubrl	4
1121	6	Gas7	4	Ugp2	4
Mbn12	6	Gnal3	4	Usp38	4
Ms4a4c	6	Gngtl	4	Usp46	4
Mtmr6	6	Golga7	4	Usp9x	4
Nlgn3	6	Gopc	4	Vmn1r7	4
Oxtr	6	Gpr149	4	Xpo7	4
Pkn2	6	Grik2	4	Ywhag	4
Ppmll	6	Hipl	4	Zdbf2	4
Rora	6	Ikzf2	4	Zfand6	4
Sdpr	6				

*Proteins predicted to be targeted by miRNAs differentially regulated by ischemia in male and female brain. The table lists show the gene name and the top number of miRNAs in each group predicted to target the gene (6–9 of 17 miRNAs in male, 4–6 miRNAs in female)*.

## Discussion

Previous studies have shown that miRNAs are regulated in the brain in response to stress, including cerebral ischemia (Fasanaro et al., 2010; Saugstad, 2010; Rink and Khanna, 2011; Liu et al., 2013; Ouyang et al., 2013). However, to our knowledge, we are the first to show miRNA responses to ischemia that are sex dependent, i.e., there are differential responses in male or female brain following ischemia. For this study we focused on those miRNAs differentially expressed between males and females greater than 1.5 *SD* from the mean ΔΔCt in order to look at the most robust changers in response to ischemia. However, mRNAs can be targeted by many miRNAs (Doench and Sharp, 2004) and even small, subtle changes in miRNA levels can lead to significant changes in mRNA translation or stability. Thus, we are currently evaluating those differentially expressed miRNAs that show a change between 1.0 and 1.5 *SD* from the mean to identify additional proteins/pathways that could underlie differential responses to ischemia in male and female brain.

Our studies also revealed a signature miRNA response to ischemia that is common to both males and females. These key findings provide a mechanism, based on differential miRNA expression in male and female brain, to identify cellular or molecular targets that could underlie the sex differences in responses to ischemia. Such insight is likely to have implications for therapeutic strategies for the treatment of stroke in men vs. women. In addition, these studies also provide the ability to examine new targets that might contribute to ischemic injury in both males and females. Thus, we are focused on identifying the cellular/molecular targets of the ischemic-regulated miRNAs to determine how these miRNAs produce differential outcomes to ischemic injury. The genes identified in this study as predicted target of the miRNAs increased by ischemia in male and female ischemic cortex (Table 2) are quite distinct, and support that these miRNAs are likely initiating changes in distinct proteins and pathways that lead to altered phenotypes in in response to ischemia in male and female brain. PANTHER pathway analysis of the gene targets of miRNAs increased by ischemia in male and female (Table 3—Supplemental Data) support that distinct pathways are induced in each gender, and future studies are focused on clarifying the importance of these pathways in differential responses to ischemia in male and female brain.

One limitation of the present studies is that miRNA target prediction is still evolving. However, we used the miRmap target prediction program (http://mirmap.ezlab.org/) to query the predicted targets of miRNAs increased by ischemia in male and female brain (Vejnar et al., 2013). The miRmap open source software library employs eleven predictor features, three of which are novel, as well as common features of target prediction including thermodynamic, evolutionary, probabilistic, and sequence-based features. This program allows the examination of feature correlations and comparison of their predictive power in an unbiased way using high throughput experimental data. Overall, target site accessibility appears to be the most predictive feature. Methods for identifying real miRNA targets are also evolving, such as using RISCtraps to stabilize and purify targets of specific miRNAs (Cambronne et al., 2012), but will be essential for future studies identifying the targets of the miRNAs regulated by ischemia in male and female brain. Another limitation is that we examined miRNA expression in the whole ischemic cortex, which may have diluted the expression of distinct miRNAs due to inclusion of both the core and penumbra. However, at 8 h reperfusion time, there are no apparent changes in cell death and thus no reliable method to indicate the boundary between core and penumbra. Thus, we will examine the regional/cellular changes in differentially expressed miRNAs to identify their potential role as mediators of cell death in the ischemic core.

In conclusion, we have shown that the sex-related differences in the response to ischemic insult observed in males and female mice is also characterized by a corresponding difference in expression levels of a specific set of miRNAs. Thus, current and future studies in our laboratory are focused on elucidating the roles of ischemia-regulated miRNAs that show distinct changes in male or female brain, along with those miRNAs that are regulated in a sex-independent fashion. We anticipate that these studies will clarify the mechanisms underlying responses to ischemia, both sex-related and non-sex-related. We also trust that they will provide guidance for the future design of therapeutic strategies to treat stroke specifically tailored to male and female patients. For example, a recent study of miRNAs in cardiac ischemia revealed that cardiomyocyte proliferation could be stimulated by the exogenous administration of miRNAs, and more importantly, that this treatment could restore cardiac mass and promote functional recovery after myocardial infarction in adult rats (Eulalio et al., 2012). We propose that future therapies could similarly be developed for cerebral ischemia, whereby administration of miRNAs designed specifically for males or females would promote functional recovery after stroke in humans.

## Author contributions

Stephanie J. Murphy and Julie A. Saugstad conceptualized the project. Yingxin Chen performed all of the mouse middle cerebral artery occlusion surgeries and brain sub-dissections. Simon J. Thompson, Catherine M. Davis, and Jennifer M. Young isolated the RNAs and performed the SABiosciences miRNA arrays. Theresa A. Lusardi and Julie A. Saugstad analyzed the miRNA array data. Jay I. Phillips isolated RNA and performed the individual qRT-PCR assays. Theresa A. Lusardi, Julie A. Saugstad, and Jay I. Phillips analyzed the miRNA qRT-PCR data. Theresa A. Lusardi, Stephanie J. Murphy, and Julie A. Saugstad prepared the manuscript.

### Conflict of interest statement

The authors declare that the research was conducted in the absence of any commercial or financial relationships that could be construed as a potential conflict of interest.
